# The complete genome sequences of *Thioglobus autotrophicus* strains EF2 and EF3, isolated from an oxycline in Effingham Inlet, British Columbia

**DOI:** 10.1128/mra.01118-23

**Published:** 2024-02-09

**Authors:** Robert M. Morris, Sayaka Mino

**Affiliations:** 1 School of Oceanography, University of Washington, Seattle, Washington, USA; 2 Laboratory of Microbiology, Faculty of Fisheries Sciences, Hokkaido University, Minato-cho, Hakodate, Japan; University of Southern California, USA

**Keywords:** isolate, SUP05, *Thioglobaceae*, complete genome

## Abstract

Here we provide the complete genome sequences of two chemoautotrophic isolates from the *Thioglobaceae* family of marine gamma-proteobacteria. The genomes were obtained from pure cultures that were initially isolated from Effingham Inlet in 2013 and revived from freezer stocks for whole genome sequencing in 2023.

## ANNOUNCEMENT

Bacteria from the *Thioglobaceae* family (SUP05 clade) often dominate marine microbial communities in anoxic environments (1), including in marine oxygen minimum zones (2–7). Identifying the genetic potential for diverse *Thioglobaceae* to carry out denitrification is critical in understanding the marine nitrogen cycle.

Strains EF2 and EF3 were isolated from Effingham Inlet at 49.093 N 125.195 W in 2013 as previously described (8, 9). Briefly, isolates were cultured by diluting cells to extinction in 96-well plates containing filter sterilized seawater media (30 kDa) amended with 1 mM thiosulfate. Plates were incubated at 10°C and wells that were positive for growth after 3 weeks were screened by 16S rRNA analyses. Glycerol stocks (10%) were prepared and stored at −80°C. Cells were revived and grown aerobically in 1 L polycarbonate bottles at 13°C, then collected onto sterile, 47 mm, 0.2 µM polycarbonate filters (MilliporeSigma, Burlington, MA) after reaching maximum cell densities (~1 × 10^6^ cells/mL). The same DNA was used for Oxford Nanopore and Illumina sequencing. DNA was extracted using a QuickGene Tissue Kit and Mini-80 system (Holliston, MA) with minor modifications. Filters were submerged in TE buffer (pH 8.0) and incubated at −80°C for 30 min and at 95°C for 10 min. Lysates were then prepared according to the instructions. The lysates were processed through QuickGene Mini-80 cartridges by loading 750 µL at a time and then washing with WDT (wash buffer). DNA was eluted in water and stored at −80°C.

We used Illumina paired end (150 bp) and Oxford Nanopore sequences to construct assemblies. Software default parameters were used unless otherwise noted. Illumina sequencing libraries were constructed using a KAPA Library Preparation Kit and sequenced on a MiSeq instrument at the Northwest Genomics Center (Seattle, WA, USA). Adapter sequences, low-quality reads (<Q30), 1 bp at the tail end, reads shorter than 20 bp, and reads that had more than 10 numbers of N bases were removed using fastp v0.23.2 (10). Nanopore libraries were constructed using a Rapid V14 barcoding kit (SQK-RBK114.24) and sequenced using a MinION Mk1C and a R10.4.1 flow cell. Basecalling was performed using Guppy v6.4.6 in accurate mode. Reads were quality filtered using NanoFilt v2.8.0 (11) with a quality threshold of 10, a length threshold of 100 bp, and trimming of 75 bp from start of each read. Assemblies were obtained using Unicycler v0.4.8 (12) with all reads (Table 1) and produced single circular contigs. Average nucleotide identity (ANI) was calculated using the ani.rb script from the enve-omics toolkit (12) and indicates that EF2 and EF3 are strains related to *T. autotrophicus* strain EF1, with ANI% scores to strain EF1 of 97.8. Genomes were annotated using the NCBI Prokaryotic Genome Annotation Pipeline (13).

**TABLE 1:** Data associated with sequencing and assembly *T. autotrophicus* EF2 and EF3

Strain	EF2	EF3
No. of raw reads		
Illumina	2,867,684	4,088,272
Nanopore	322,030	424,316
Nanopore raw read N_50_ (bp)	6,124	6,923
Assembled contig (bp)	1,497,563	1,442,386
Coverage	828 ×	1340 ×
Rotation	dnaA start	dnaA start
GC (%)	39.1	39.4
Genes	1,623	1,591
Protein-coding genes	1,573	1,545

## Data Availability

Genome sequences were deposited in GenBank under BioSample, BioProject, Sequence Read Archive, and Genome assembly accession numbers: Strain EF2, SAMN37983939, PRJNA1032126, SRR26639703, SRR27349087, SRR27349088, and CP137632; Strain EF3, SAMN37983940, PRJNA1032127, SRR26660965, SRR27348713, and CP137633.
